# Supplementary material for: Medicinal Properties of Anchusa strigosa and Its Active Compounds
Source: Molecules. 2022 Nov 25;27(23):8239. doi: 10.3390/molecules27238239 (PMC9741094; doi:10.3390/molecules27238239)

GC-MS and LC-ESI-MS chromatograms of extracts of *Anchusa strigosa*

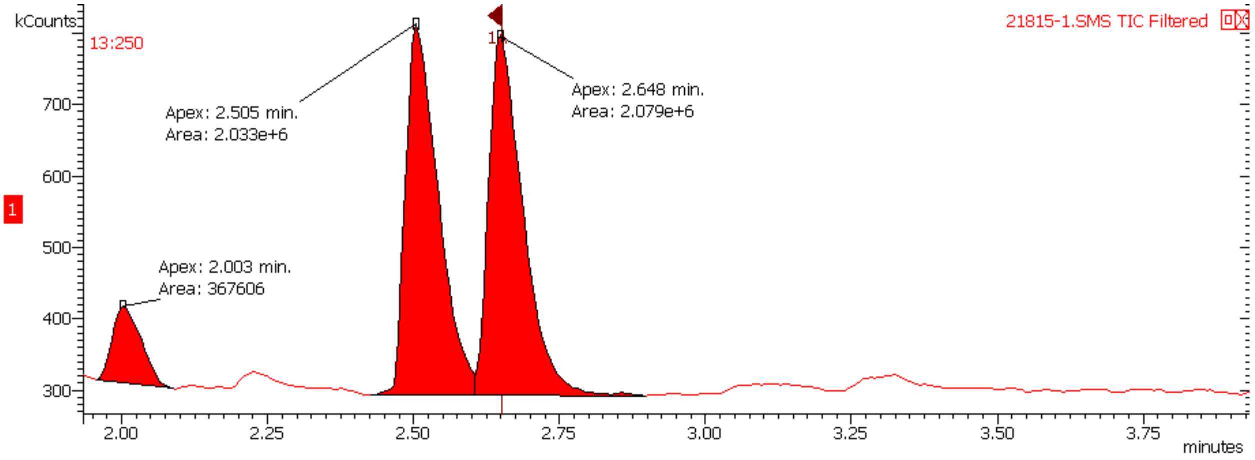

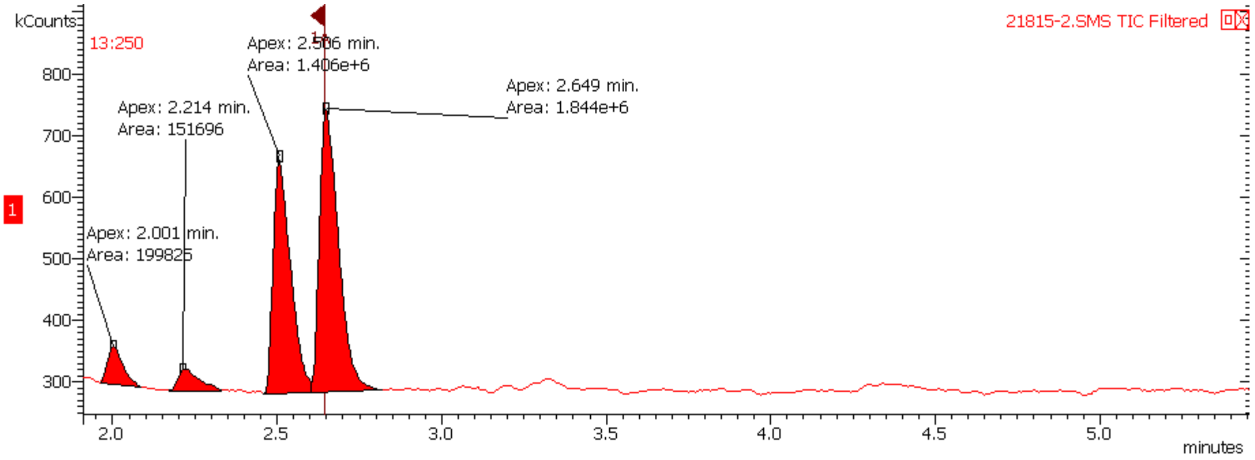

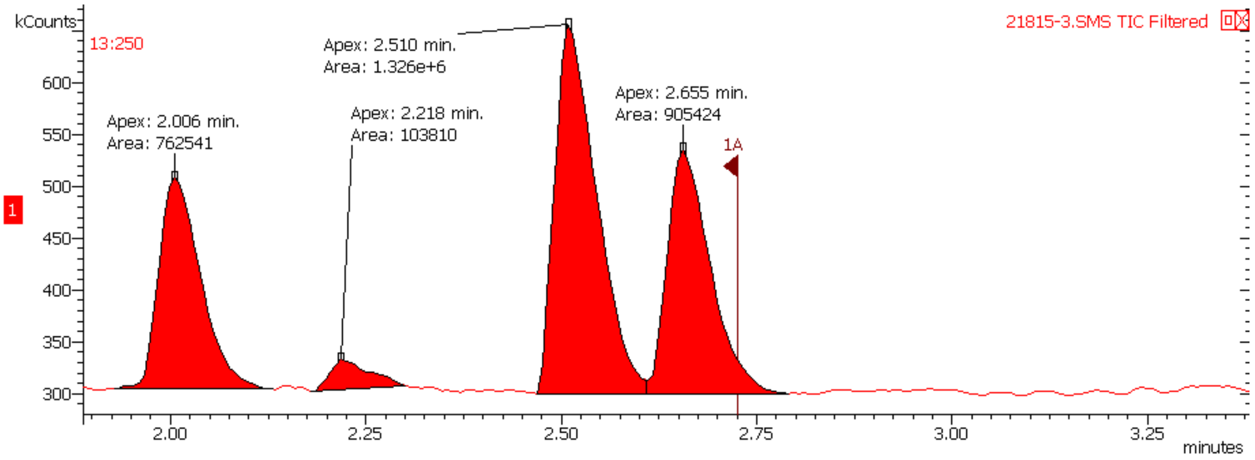

GC-MS and LC-ESI-MS chromatograms of extracts of *Anchusa strigosa*

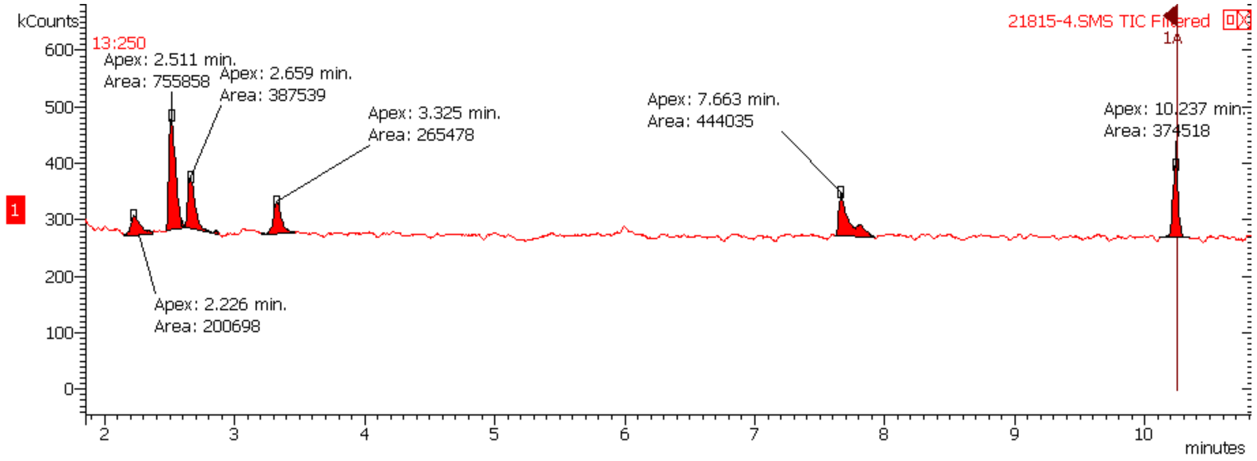

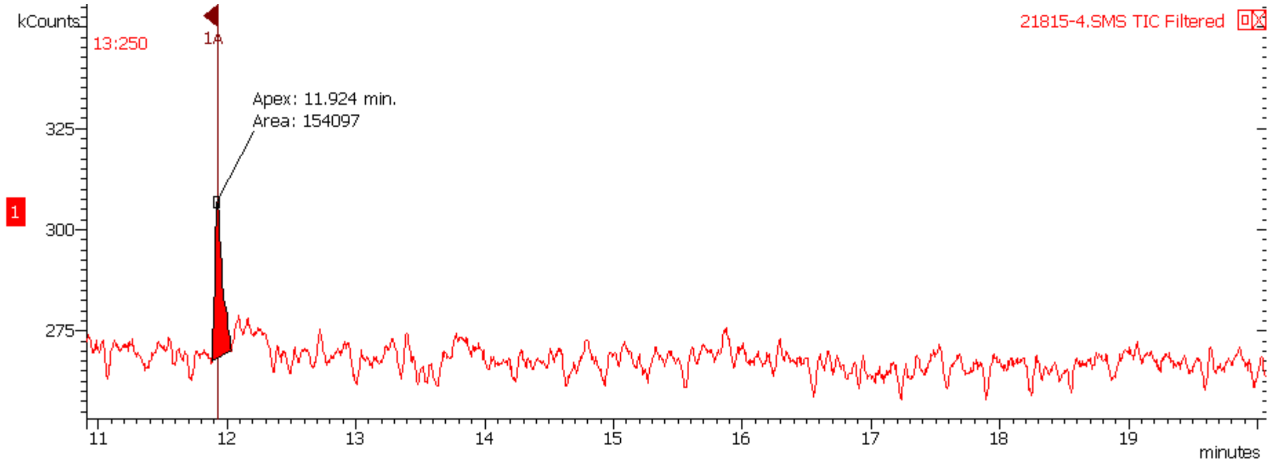

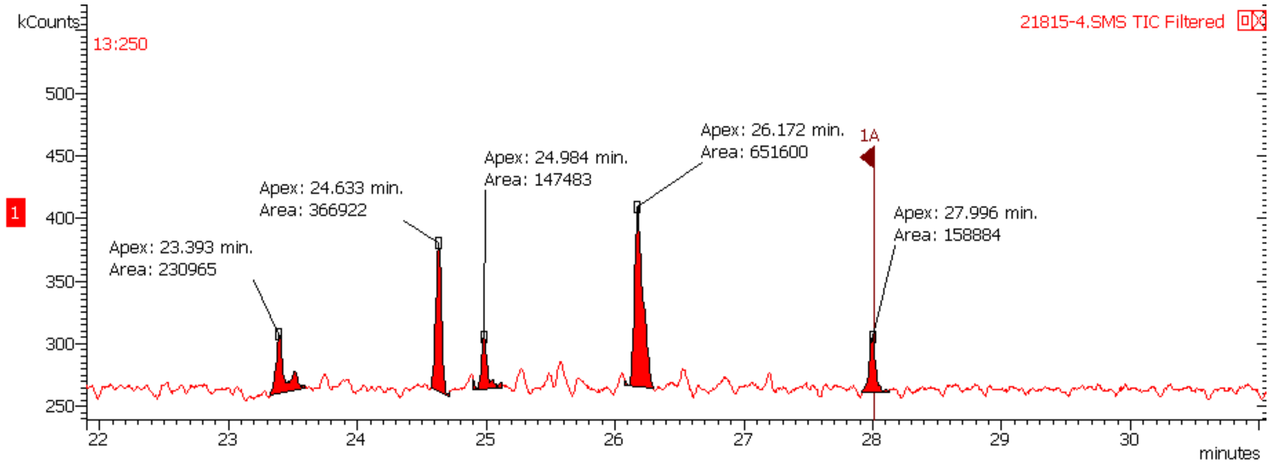

GC-MS and LC-ESI-MS chromatograms of extracts of *Anchusa strigosa*

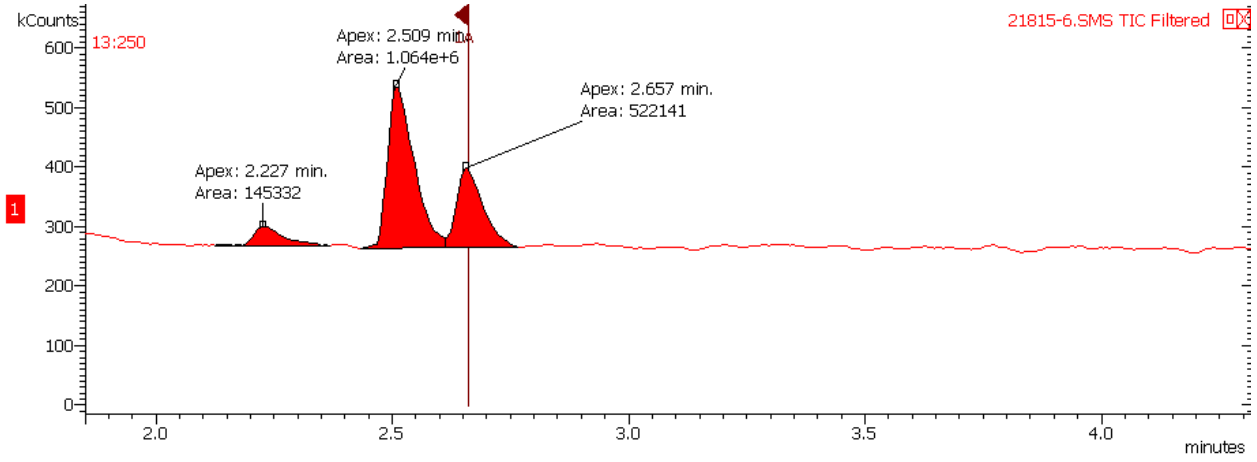

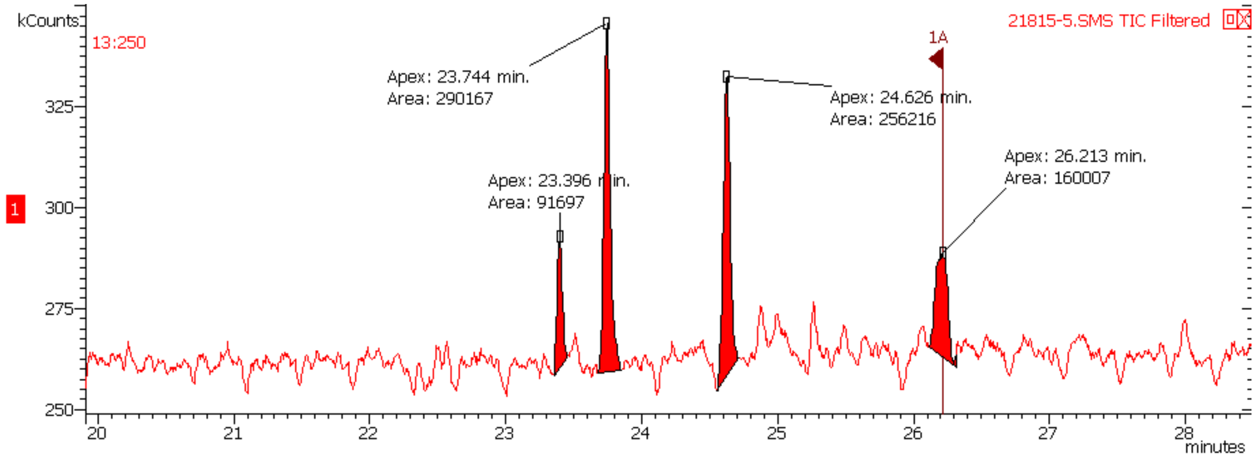

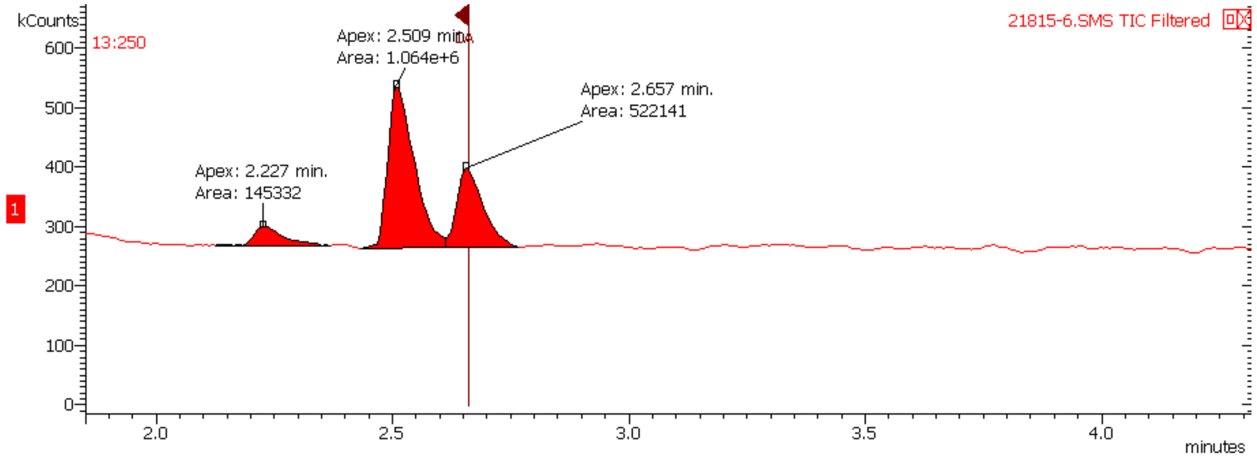

GC-MS and LC-ESI-MS chromatograms of extracts of *Anchusa strigosa*

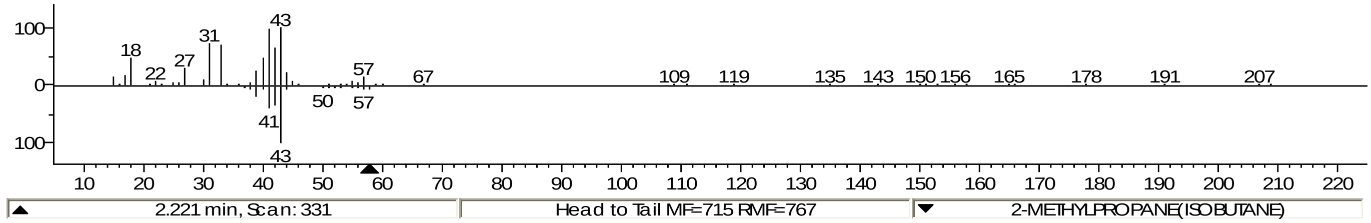

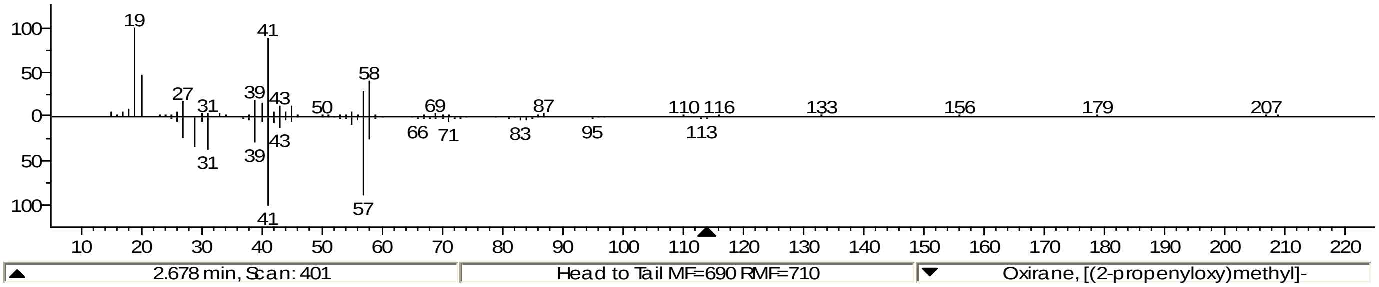

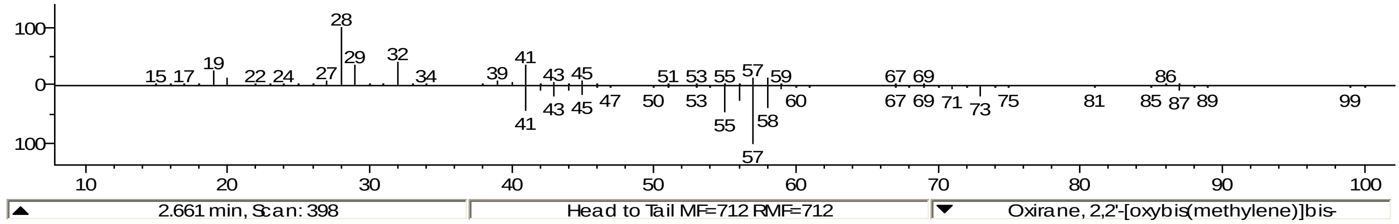

GC-MS and LC-ESI-MS chromatograms of extracts of *Anchusa strigosa*

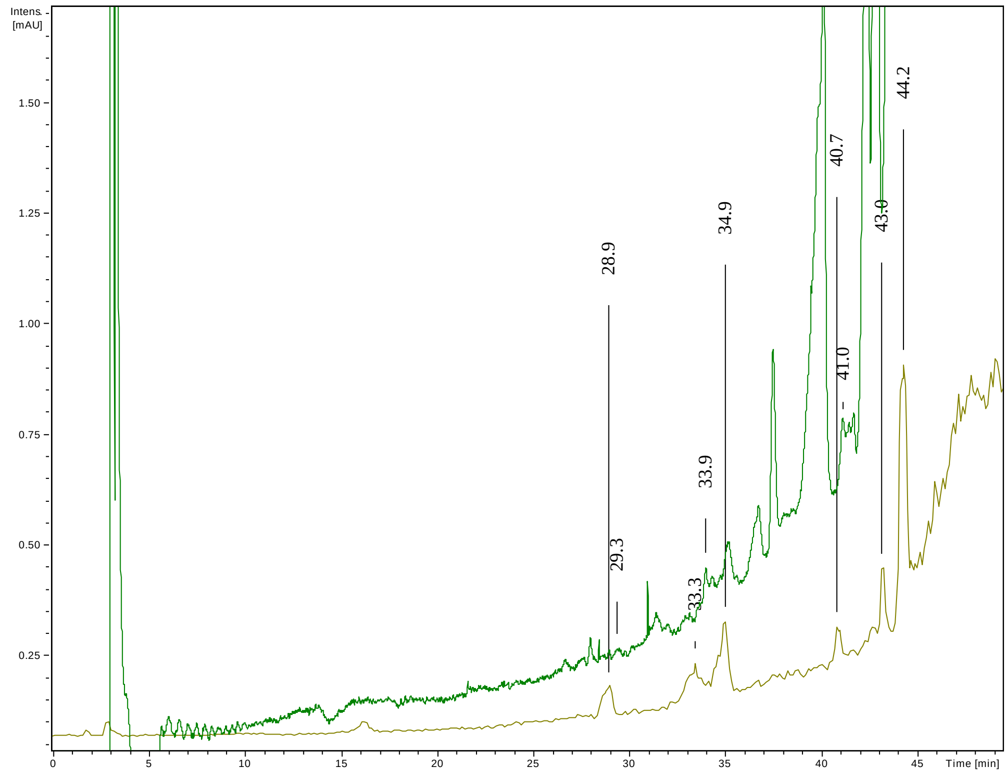

UV Chromatogram -360nm
TIC. MS. ESI. Positive Mode. Target mass 500.

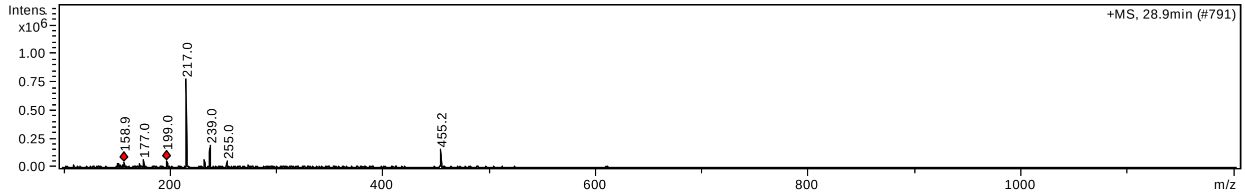

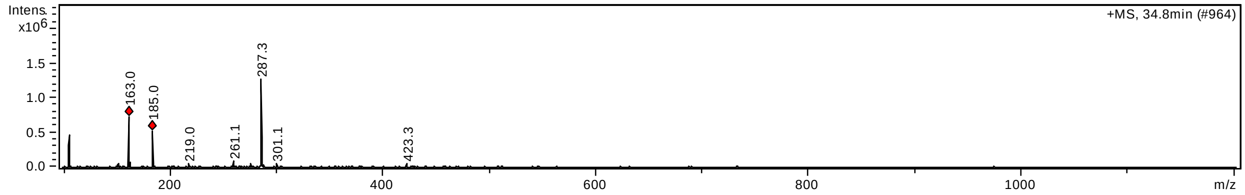

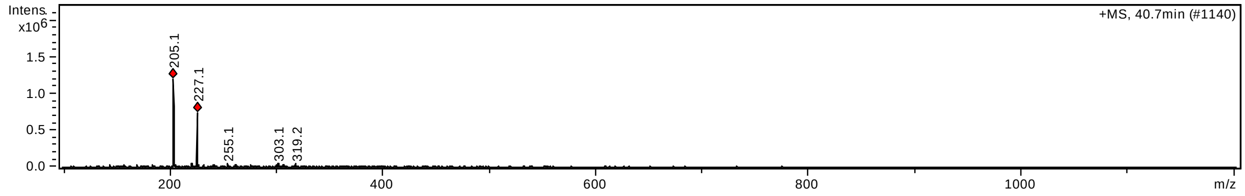

GC-MS and LC-ESI-MS chromatograms of extracts of *Anchusa strigosa*

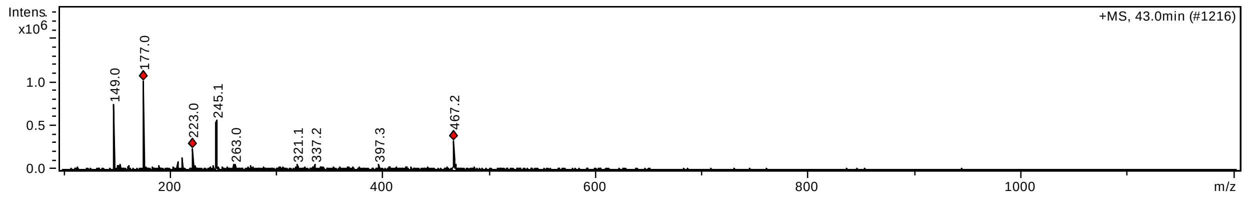

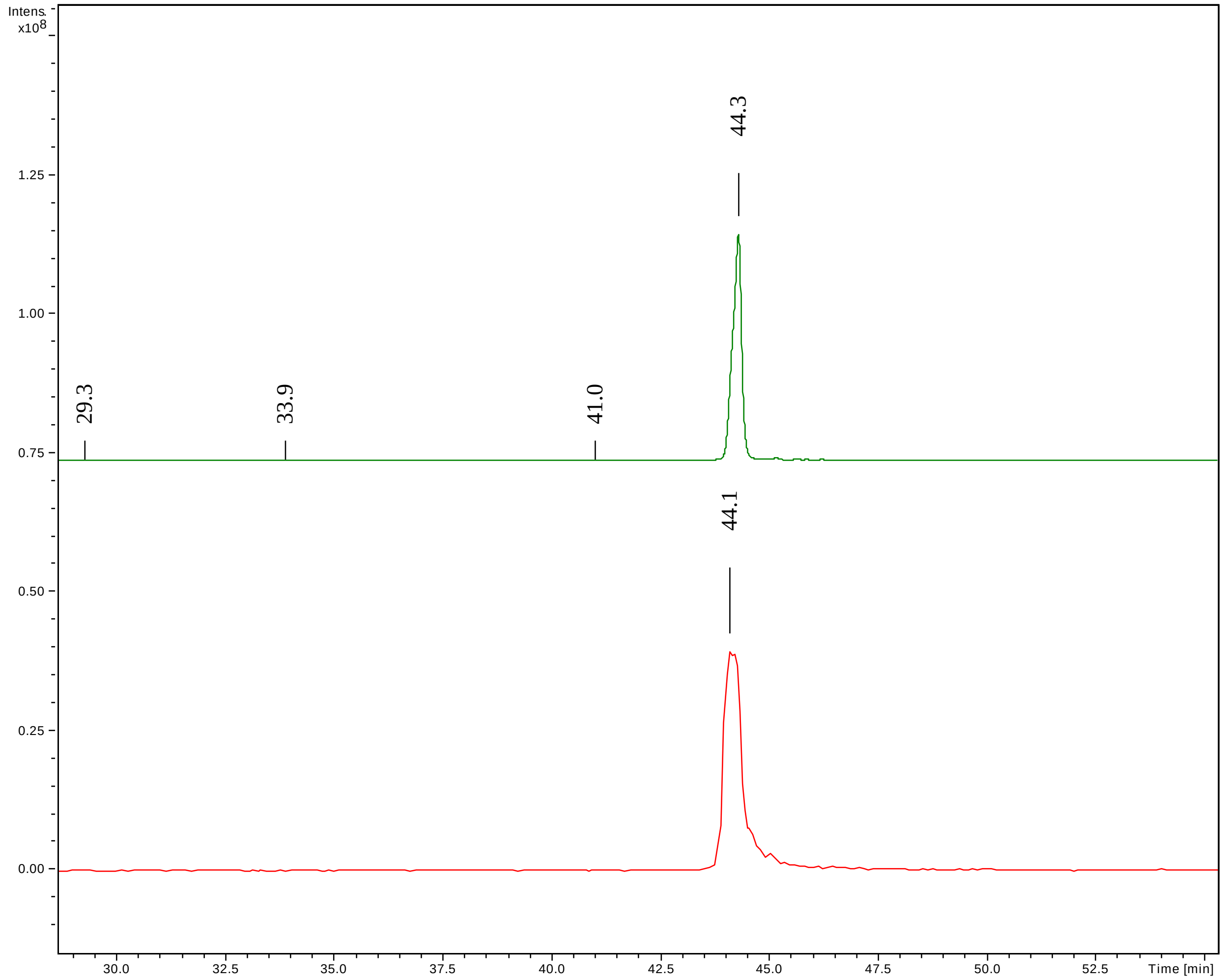

UV Chromatogram -360nm

EIC. MS. ESI. Positive Mode. Target mass 500.

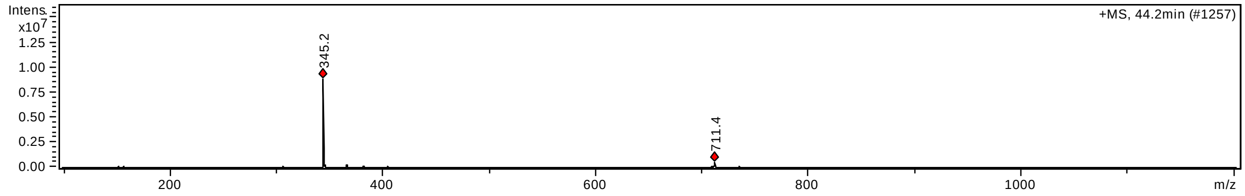

GC-MS and LC-ESI-MS chromatograms of extracts of *Anchusa strigosa*

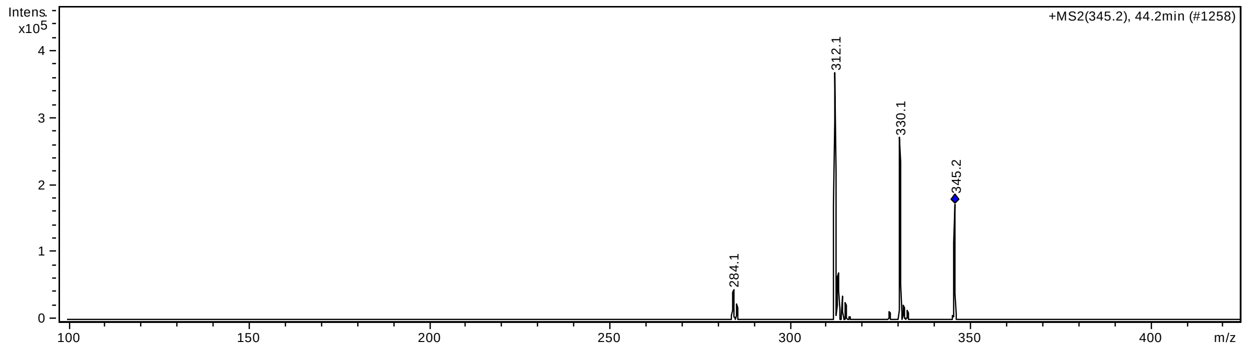

Supplement: Supplementary file 1 [file molecules-27-08239-s001.zip › molecules-2023978-supplementary.pdf]
